# Joint-Angle Coordination Patterns Ensure Stabilization of a Body-Plus-Tool System in Point-to-Point Movements with a Rod

**DOI:** 10.3389/fpsyg.2016.00826

**Published:** 2016-06-03

**Authors:** Tim A. Valk, Leonora J. Mouton, Raoul M. Bongers

**Affiliations:** Center for Human Movement Sciences, University of Groningen, University Medical Center GroningenGroningen, Netherlands

**Keywords:** tool use, uncontrolled manifold (UCM), joint-angle variability, end-effector kinematics, joint-angle trajectories, point-to-point movements

## Abstract

When performing a goal-directed action with a tool, it is generally assumed that the point of control of the action system is displaced from the hand to the tool, implying that body and tool function as one system. Studies of how actions with tools are performed have been limited to studying either end-effector kinematics or joint-angle coordination patterns. Because joint-angle coordination patterns affect end-effector kinematics, the current study examined them together, with the aim of revealing how body and tool function as one system. Seated participants made point-to-point movements with their index finger, and with rods of 10, 20, and 30 cm attached to their index finger. Start point and target were presented on a table in front of them, and in half of the conditions a participant displacement compensated for rod length. Results revealed that the kinematics of the rod's tip showed higher peak velocity, longer deceleration time, and more curvature with longer rods. End-effector movements were more curved in the horizontal plane when participants were not displaced. Joint-angle trajectories were similar across rod lengths when participants were displaced, whereas more extreme joint-angles were used with longer rods when participants were not displaced. Furthermore, in every condition the end-effector was stabilized to a similar extent; both variability in joint-angle coordination patterns that affected end-effector position and variability that did not affect end-effector position increased in a similar way vis-à-vis rod length. Moreover, the increase was higher in those conditions, in which participants were not displaced. This suggests that during tool use, body and tool are united in a single system so as to stabilize the end-effector kinematics in a similar way that is independent of tool length. In addition, the properties of the actual trajectory of the end-effector, as well as the actual joint-angles used, depend on the length of the tool and the specifics of the task.

## Introduction

A tool in use is “a sort of extension of the hand, almost an attachment to it or a part of the user's own body” (Gibson, 2015, p. 35). This suggests that when using tools, body and tool function as one system, resulting in a shift of the end-effector from the hand to the tool. This shift has been implied in studies focusing on end-effector kinematics of tool use (Heuer and Sülzenbrück, 2009; Jacobs et al., 2009). For instance, Heuer and Sülzenbrück (2009) showed that in learning to move the tip of a complex tool between targets, the movement trajectories of the hand went from straight to curved, whereas the trajectories of the tool's tip went from curved to straight. This implied that, when learning, the trajectory of the tip of the tool gradually changed, so that the tool's tip was moving more as the hand moved in non-tool movements. Others have concentrated on processes underlying tooling kinematics; Van der Steen and Bongers (2011) studied participants who were making point-to-point movements with the tip of a small rod attached to the index finger. They showed that the joint-angle coordination patterns in the arm stabilized the rod's tip and the hand to a similar extent. Given that stabilized variables are variables that are controlled (cf. Schöner, 1995), this indicates that the tip of the tool is also controlled during tool use. Until now, end-effector kinematics and their stabilization through joint-angles in the arm during tool use have not been studied together. However, they are intrinsically coupled, because end-effector kinematics follows from joint-angle coordination patterns in the arm. Importantly, the resulting movement, that is, end-effector kinematics as well as joint-angles, is influenced by the torques resulting from moving the rod. Therefore, the current paper has investigated how end-effector kinematics and the stabilizing properties of joint-angle coordination patterns are affected by tool length in order to advance our understanding of how body and tool function as one system. This was done by studying able-bodied participants making point-to-point movements with rods of varying lengths attached to the index finger.

When a rod is used to perform a point-to-point movement, properties of the rod, such as its length and mass, affect the way the point-to-point movement is made. For instance, trajectories of the tip of the end-effector in an aiming task in 2-D space, using a manipulandum (i.e., an apparatus which consists of a freely rotating handle with a pointer attached to it, mounted on one end of a two-link chain with two pivots), exhibited larger curvatures, larger peak velocities, and shorter movement times when participants used longer pointers (Dean and Brüwer, 1997). In addition, movement times of point-to-point movements in 3-D space, using short probes, increased as probe lengths increased (Baird et al., 2002). These findings showed that tool length affected the kinematics of point-to-point movements vis-à-vis the tool's tip. Note that, for variations in tool length, not only end-point kinematics but also joint-angles in the arm may be affected. That is, a similar change in one of the joint-angles in the arm has a greater effect on end-effector movement for longer tools (cf. Cruse et al., 1990; Dean and Brüwer, 1997). This means that studying how tool length affects end-effector kinematics and arm joint-angle coordination patterns is not trivial in our understanding of how body and tool function together, and this is therefore the focus of the current paper.

This present study was a follow-up on an earlier, above-mentioned study that focused on joint-angle coordination in rod reaching (Van der Steen and Bongers, 2011). In that previous study, participants had to make point-to-point movements with their index finger and with rods of 10, 20, and 30 cm attached to the index finger. During these movements, participants were displaced from the target at a distance equal to the length of the rod, with which they performed the movement. For instance, if participants made movements with a rod of 10 cm, they were displaced 10 cm backwards. It was found that only the flexion-extension angle in the elbow, and the abduction-adduction angle in the wrist differed across rod lengths. Moreover, the coordination between the joints in the arm (e.g., covariation between joints to stabilize the movement of the rod's tip) was independent of rod length. Although, these results support the idea that the body and tool function as one system, the similarity in joint-angle coordination patterns across rod lengths might have been engendered by the displacement of the participant during the pointing movements (cf. Van der Steen and Bongers, 2011). In other words, the possible effects of rod length could have been counterbalanced by the participants' displacement from the target. To be able to assess the effect of participant displacement, the current study measured not only situations in which the participants were displaced from the target, but also situations in which participants remained at the same distance to the target, while rod length was varied.

To examine how joint-angle coordination patterns varied when rods of different length were used, we employed the uncontrolled manifold (UCM) method (Schöner, 1995; Scholz and Schöner, 1999; Latash et al., 2007). This method exploits the fact that, because of the abundant degrees of freedom in the arm, a wide variety of arm configurations can be used, while keeping the tip of the end-effector at the same spot in 3-D space. In other words, the position of the end-effector tip in 3-D space can be stabilized at the same position, while the values of the joint-angles in the arm vary. These varying configurations of joint-angles, resulting in the same end-effector position, can be represented by a manifold in a joint space that has an axis involved for each joint. The UCM method divides the total variability in joint-angles over repetitions of movements in a part that varies along this manifold, and thus provides for goal-equivalent solutions (Goal-Equivalent Variability; GEV) and a part that varies orthogonally to this manifold, and thus results in different end-effector positions (Non-Goal-Equivalent Variability; NGEV). If the value of GEV exceeds the value of NGEV, it is assumed that the end-effector position is stabilized. Several studies have applied the UCM method successfully in reaching and pointing tasks (Scholz et al., 2000; Domkin et al., 2002, 2005; Tseng et al., 2002, 2003; Van der Steen and Bongers, 2011; Kim et al., 2012; Krüger et al., 2012; Verrel et al., 2012; Rein et al., 2013; Greve et al., 2015), showing that the end-effector position, usually the tip of the index finger, was stabilized during the movement.

In the current study, the participants made pointing movements with rods attached to their index finger. In half of the conditions the increased end-effector length was compensated for by a displacement of the participant with respect to the target, whereas in the other half no compensation for rod length was given. The question posed by the current study was how the body and tool function as one system. Therefore, we studied how the kinematics of the rod's tip as well as the degree of stabilization of the tip were affected by rod length. To do this, we examined how the combination of participant placement and different end-effector lengths was affecting: (1) the trajectory of the tip of the end-effector, (2) joint-angle trajectories, (3) joint-angle coordination patterns, and (4) the relationship between end-effector kinematics and joint-angle coordination patterns. The conditions with participant displacement replicated those of Van der Steen and Bongers (2011), and from that we expected small effects from rod length on joint-angle trajectories, and no effect of rod length on the stabilization of the rod's tip, in these conditions. The analyses of the kinematics of the end-effector and conditions without participant displacement were novel; we expected that the use of longer rods would result in decreased target accuracy, increased movement times, larger curvatures, and higher peak velocities (Dean and Brüwer, 1997; Baird et al., 2002). Following on the idea that body and tool function as one system, we hypothesized that participants' displacement would not result in large effects on end-effector kinematics. With regard to joint-angle trajectories, we expected that different joint-angle trajectories would be used for the different tool lengths, when participants were not displaced with respect to the target, based on previous studies (Cruse et al., 1990; Dean and Brüwer, 1997).

Finally, with respect to the joint-angle coordination patterns, we expected that in all conditions the end-effector kinematics would be stabilized, that is, finding larger values for GEV than for NGEV. However, in some conditions, especially when the participants were not displaced and the rod was long, the joint-angle configurations used to control the rod might not be in the range of what is regularly used in controlling a tool. Therefore, we expected the non-stabilizing variability, that is, NGEV, to increase in these conditions. In addition, we expected that GEV would also increase in these conditions to ensure stabilization of the tip of the tool (cf. Greve et al., 2015), which would reflect a way in which body and tool function as one system.

## Methods

### Participants

Seven male (mean [SD] age: 21.29 [1.38] years) and eight female (20.50 [1.77] years) participants, 15 in total, made pointing movements in a forward direction. All participants were right-handed, had no health issues, and had normal or corrected-to-normal visual sight. Participants received verbal and written information concerning the procedures during the experiment and signed an informed consent in advance of the experiment.

### Ethical statement

The ethics committee of the Center of Human Movement Sciences, University Medical Center Groningen gave their consent for conducting the current study, based on the principles expressed in the Declaration of Helsinki. Before the start of the experiment, each participant read and signed a written informed consent.

### Experimental set-up

**Figure 1:**
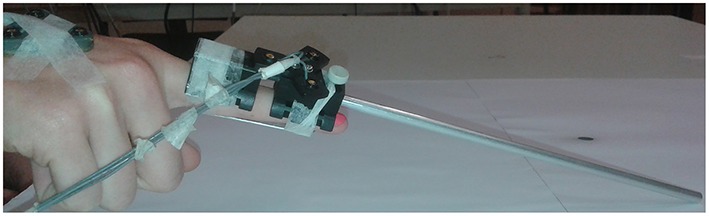
**Splinted index finger of participant with aluminum holder for rod attachment**. The index finger was splinted by means of a small aluminum plate on the ventral side of the digit.

The pointing movements were measured with an Optotrak 3020 system (Northern Digital, Waterloo, Ontario, Canada), using two units that were sampling at 100 Hz. To record the pointing movements, five triangular rigid bodies were fixed onto the right side of the participant's body, following the example of Van Andel et al. (2008): the first was attached to the sternum, the second to the flat part of the acromion, the third laterally to the upper arm just below the insertion of the deltoid, the fourth laterally to the lower arm just proximal to the ulnar and radial styloids, and the fifth to the dorsal surface of the hand. The rigid bodies were made of hard PVC and contained an Optotrak LED in each corner of the triangle. Two rigid bodies, attached to the sternum and the upper arm, had a leg length of 6 cm. The other rigid bodies had a leg length of 4 cm. An extra set of three Optotrak LEDs was placed on an aluminum holder that was used to attach the rods to the index finger (Figure 1). To link the positions of the markers to the anatomical positions of the participants, 18 bony landmarks of the participants were digitized using a pointer device (Van Andel et al., 2008).

The rods used during this experiment were made of aluminum; had a length of 10, 20, and 30 cm; a diameter of 0.5 cm; and a mass of 8, 16, and 24 g, respectively. As mentioned above, the rods were attached to the index finger with an aluminum holder (Figure 1), which had a mass of 50 g. The holder was attached on the dorsal side of the index finger with tape. The index finger was splinted on the ventral side with a small aluminum plate (Figure 1) to prevent movement of the interphalangeal joints but allowing free motion of the metacarpophalangeal joint.

The start and target points of the pointing movements had a diameter of 1 cm and were located 30 cm apart from one another on a fixed position on the table in front of the participants.

### Design

The experiment involved eight experimental conditions, based on two kinds of participant placement (displacement or no displacement) and four different end-effector lengths. In those four conditions in which the participant was displaced, the distance of the participant to the target was adjusted according to the length of the rod. For example, when participants had to make pointing movements with a rod of 10 cm, the chair of the participant was placed 10 cm further away from the target than in conditions, in which participants had to make pointing movements with their index finger. In the other four conditions, the distance of the chair to the table was not adjusted for rod length. In all eight conditions, participants had to perform 25 trials of pointing movements. The blocks of 25 trials were presented in a random order that differed for each participant.

### Experimental procedure

The participants sat in a chair with an extended back while making the pointing movements. During these movements, the trunk of the participants was strapped to the back of the chair in such a way that the trunk was prevented from contributing to the pointing movements but ensuring that the shoulder joint was not hindered by this constraint. To make sure that movements had a correct starting position, first, the position of the participant with respect to the target for the condition, in which the index finger had to be used, was determined. At the start of each new block of trials the position of the participant with respect to the target was changed, depending on the new condition. Furthermore, a similar starting position of the arm over trials was assured by means of an elbow placer, positioned on the right side of the participant. The position and the height of this placer was adjusted to the height of the participant's olecranon, while participants placed the tip of their index finger or the tip of the rod at the starting point of the movement, sitting in a comfortable manner.

At the beginning of each trial, the participants sat in the chair with an initial configuration of the upper arm next to the body and the elbow resting on the placer. The tip of the index finger or the tip of the rod was placed at the starting position. The participants sat comfortably during this initial posture. After a random time interval, the examiner gave the starting signal, after which the participants reached as quickly and accurately as possible for the target. The trial ended with a second signal by the examiner, after the participant held the index finger or tip of the rod on the target for a short period. The aluminum holder, in which the various rod were inserted, continued to be attached to the index finger, whether the participant used a rod to point or not.

### Computation of joint-angles

The following joint-angles were examined during this study: shoulder plane of elevation, shoulder angle elevation, shoulder endorotation-exorotation, elbow flexion-extension, forearm pronation-supination, wrist abduction-adduction, wrist flexion-extension, finger abduction-adduction, and finger flexion-extension. These joint-angles were computed following ISB guidelines for the upper extremity (Wu et al., 2005). Segment orientations, both to the global frame of reference and relative to each other, were calculated using the local coordinate systems from the digitized bony landmarks and the displacement of the markers at the rigid bodies. The rigid body on the holder was used to calculate end-effector movement.

### Computation of UCM variables

The UCM method (Schöner, 1995; Scholz and Schöner, 1999; Latash et al., 2007), as detailed by Yen and Chang (2010) and Verrel (2011), was used to assess the joint-angle coordination patterns. In this method, the 3-D position of the tip of the end-effector (i.e., the tip of the index finger or the tip of the rod) was selected as performance variable, whereas the nine examined joint-angles were selected as elemental variables. To relate changes of the elemental variables to changes in the performance variable, the Jacobian matrix (J) was computed (Domkin et al., 2005). The elements of this matrix contained the partial derivatives of the positional coordinates of the performance variable, with respect to the mean joint-angle configuration. The null space of J represents the changes in elemental variables that do not lead to a change in the performance variable, whereas the orthogonal component of J represents the changes in elemental variables that do lead to a change in the performance variable.

Per condition, a covariance matrix C of the joint-angle trajectories across trials was computed. Based on this matrix, the UCM variables GEV, NGEV, and V_Ratio_ were computed following:

(1) $$\text{NGEV}=\text{trace}(\text{orth}{\left({\text{J}}^{\text{T}}\right)}^{\text{T}}{}^{*}{\text{C}}^{*}\text{orth}({\text{J}}^{\text{T}}))\text{/d}$$

(2) $$\text{GEV}=\text{trace}(\text{null}{\left(\text{J}\right)}^{\text{T}}{}^{*}{\text{C}}^{*}\text{null}(\text{J}))/\text{n}-\text{d}$$

(3) $${\text{V}}_{\text{Ratio}}\;=\frac{\text{GEV}}{\text{NGEV}}$$

In this, the total variability in elemental variables is projected to the null space of J (null(J)^T^) to compute GEV, and to the orthogonal space of J (orth(J^T^)^T^) to compute NGEV. Furthermore, n denotes the dimension of the joint space (*n* = 9) and d the dimension of the performance space (*d* = 3). As can be seen in the equations above, each UCM variable is normalized by the dimension of the relevant space.

In general, the UCM method, as outlined above, results in a non-normal distribution of the data. Therefore, before statistical analysis, all UCM variables were corrected for this non-normality using a logarithmic transform (Verrel, 2010), resulting in GEV_Log_ = log(GEV), NGEV_Log_ = log(NGEV) and V_RatioLog_ = log(V_Ratio_). When GEV_Log_ > NGEV_Log_, and thus V_RatioLog_ > 0, the performance variable, that is, the 3-D position of the tip of the end-effector is controlled using flexible joint-angle coordination patterns.

### Data analysis

The raw positional data from the Optotrak markers were processed, using custom-made MATLAB (The Mathworks Inc., MA, USA, version 2010a) programs. Accuracy of the movements at the target was determined by computing the absolute and variable error at the end of the movement. The absolute error was computed as the difference between the position of the tip of the end-effector at the end of the movement and the center of the target, and was averaged over the trials for every condition. The variable error for every condition was computed as the standard deviation of the absolute error over trials within a condition.

The start and end of the movement were defined as the moment when tangential velocity of the end-effector went above or below a speed of 25 mm/s, respectively, and the position of the pointer tip was outside or within a radius of 10 mm of the start point or target, respectively. To compute the tangential velocity of the end-effector, first, the derivatives of the 3-D positional data along each axis were calculated. Subsequently, the tangential velocity was computed by taking the square root of the sum of the squared derivatives of the 3-D positional data of the end-effector.

For the period between start and end of the movement, several kinematic variables were computed. First, the maximum reach velocity was defined as the maximum tangential velocity of the end-effector during the movement. Acceleration and deceleration time were defined as the time between the start or end of the movement, and the moment of maximum reach velocity, respectively. Horizontal curvature was defined as the maximum deviation of the end-effector in the transversal plane from a straight line between starting point and target. Similarly, vertical curvature was defined as the maximum deviation of the end-effector in the sagittal plane from a straight line between starting point and target.

For the UCM analysis, each trial was time-normalized. After this normalization, the UCM variables at 100%—that is, at the end point—of the movement were selected for statistical analysis. This was done because, in essence, only at this moment, the tip of the end-effector of the body-plus-tool system needs to be stabilized by joint-angle coordination patterns. Five instants during the movement (at 1, 25, 50, 75, and 100%) were selected for the statistical analysis of joint-angle trajectories.

### Statistical analysis

Several repeated measures ANOVAs were performed to statistically analyze the processed data. Two-way repeated measures ANOVAs with rod length (0, 10, 20, and 30 cm) and participant placement (displaced or not), as within subject factors, were used to analyze the acceleration time, deceleration time, maximum reach velocity, horizontal curvature, vertical curvature, GEV_Log_, NGEV_Log_, and V_RatioLog_. Joint-angle trajectories of individual joint-angles were analyzed, using three-way repeated measures ANOVAs with rod length, participant placement, and movement instant (1, 25, 50, 75, and 100%) as within subject factors.

If, within these repeated measures ANOVAs, the assumption of sphericity was violated, the Greenhouse-Geisser correction was used. For the interpretation of the effects of the ANOVAs, the generalized eta-squared ($\eta_{\text{G}}^{2}$) for effect sizes was used (Olejnik and Algina, 2003; Bakeman, 2005). The interpretation of these effect sizes was done by using Cohen (Cohen, 1988, pp. 413–414), who recommended interpreting an effect size of 0.02 as a small effect, an effect size of 0.13 as a medium effect, and an effect size of 0.26 as a large effect (see also Bakeman, 2005). We used Bonferroni correction when multiple *post-hoc* tests were performed. In a Bonferroni adjusted pairwise comparison performed in this way using SPSS, the *p*-value is adjusted by multiplying the unadjusted *p*-value by the number of contrasts being made. Thus, significant mean differences in consideration of this adjustment are still represented as *p* < 0.05. All significant effects with an effect size smaller than 0.02 were excluded from the results. The analyses were performed using SPSS version 17.

## Results

### Target accuracy

**Figure 2:**
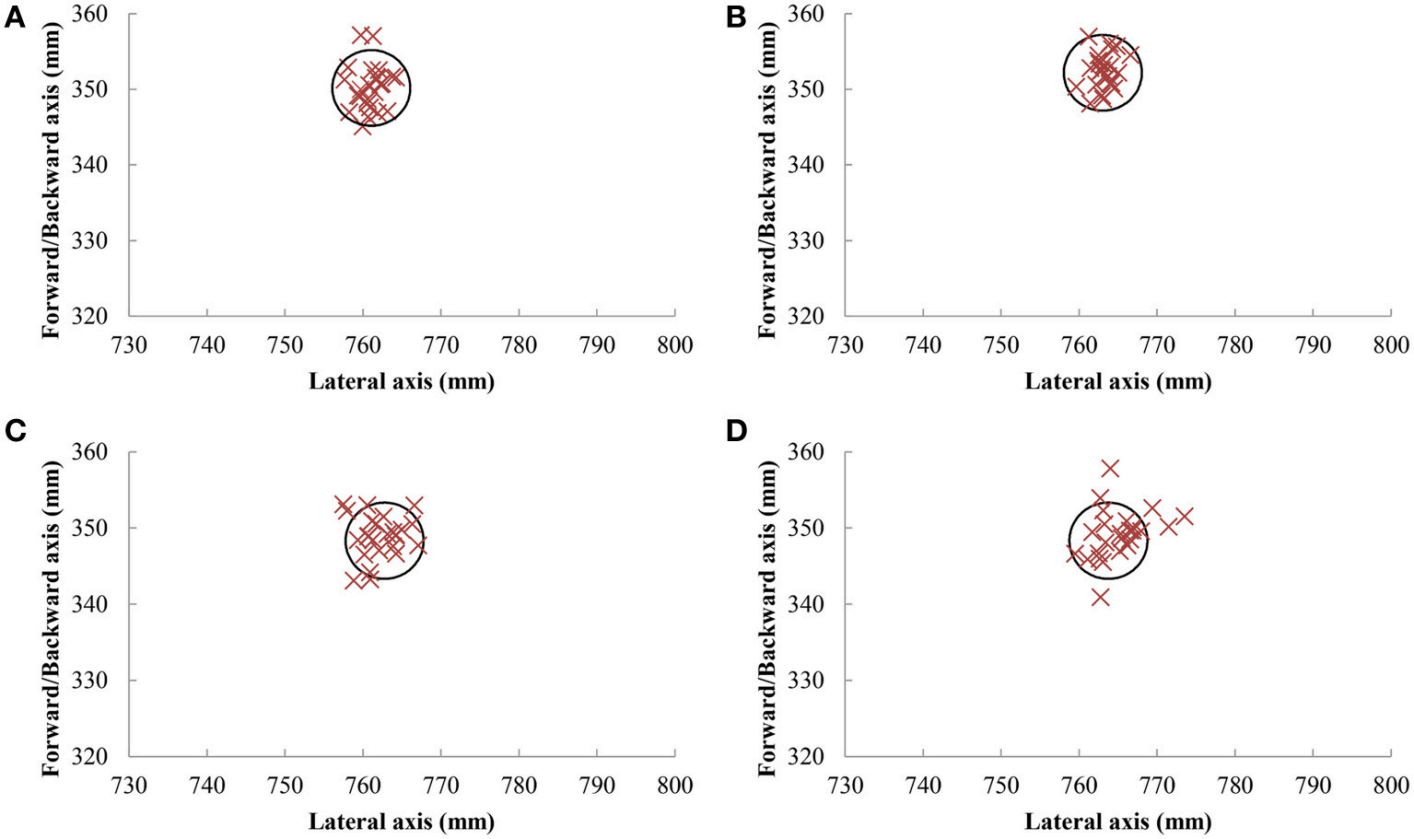
**Accuracy at the target of one participant performing typical behavior**. Accuracy of pointing movements with the index finger with and without participant displacement are shown in panels **(A,B)**, respectively, whereas pointing movements with a rod of 30 cm with and without participant displacement are presented in panels **(C,D)**, respectively.

The target accuracy of one typical participant is shown in Figure 2. The target accuracy was generally high in all the conditions (Table 1). This was also demonstrated by the fact that the largest deviation from the target was 7.6 mm, across all participants. Furthermore, participants were consistent in their accuracy, with a maximum variable error of 3.8 mm. From these values, we concluded that participants performed the task as requested.

**Table 1:** **Means (SD) for absolute and variable errors**.

**Rod length**	**No participant displacement**		**Participant displacement**	
	**Absolute error (mm)**	**Variable error (mm)**	**Absolute error (mm)**	**Variable error (mm)**
0 cm	4.7 (2.6)	3.0 (1.0)	5.4 (2.5)	2.8 (0.9)
10 cm	4.9 (3.0)	2.8 (1.4)	4.3 (2.3)	2.7 (1.4)
20 cm	5.9 (3.4)	3.0 (1.1)	5.2 (3.8)	3.1 (1.6)
30 cm	5.5 (2.7)	3.6 (1.6)	5.1 (3.1)	3.6 (1.6)

### End-point kinematics

All kinematic variables, with the exception of acceleration time, gradually increased when participants pointed with longer end-effectors (Table 2). In other words, for movements with longer rods, a higher peak velocity and a longer deceleration time were found, and movements were more curved in the horizontal plane as well as in the vertical plane (i.e., longer rods were lifted higher). *Post-hoc* comparisons revealed that movements differed significantly across all rod lengths in deceleration time and vertical curvature (all *p*'s < 0.05). Furthermore, *post-hoc* comparisons showed that horizontal curvature differed significantly across rod lengths, with the exception of the difference between movements with the index finger and movements with a rod of 10 cm (all *p*'s ≤ 0.01). The maximum reach velocity of movements differed only between movements with rods of 10 and 20 cm, and movements with rods of 10 and 30 cm (both *p*'s < 0.05).

**Table 2:** **Significant main and interaction effects for kinematic variables**.

**Dependent variable**	**Within-subject factor**		**Mean**	***SD***	***F***	***df***	***p***	***$\eta_{\text{G}}^{2}$***
**DECELERATION TIME (S)**								
	Rod length	0 cm	0.41	0.14	53.26	3, 42	<0.001	0.14
		10 cm	0.47	0.13				
		20 cm	0.54	0.18				
		30 cm	0.57	0.17				
**MAXIMUM REACH VELOCITY (M/S)**								
	Rod length	0 cm	1.06	0.29	5.69	3, 42	<0.05	0.02
		10 cm	1.05	0.30				
		20 cm	1.12	0.35				
		30 cm	1.16	0.39				
**HORIZONTAL CURVATURE (MM)**								
	Rod length	0 cm	11.44	2.75	68.69	3, 42	<0.001	0.55
		10 cm	13.04	3.10				
		20 cm	16.48	3.69				
		30 cm	21.02	4.57				
	Participant displacement	No	16.27	5.66	7.46	1, 14	<0.05	0.05
		Yes	14.72	4.43				
	Rod length × Participant displacement				3.97	3, 42	<0.05	0.05
**VERTICAL CURVATURE (MM)**								
	Rod length	0 cm	48.89	17.79	57.70	3, 42	<0.001	0.44
		10 cm	62.02	13.55				
		20 cm	73.01	13.24				
		30 cm	83.02	14.23				

Two effects of participant placement on horizontal curvature (main effect and interaction with rod length) proved significant (Table 2). That is, pointing movements were more curved in the horizontal plane when the participant was not displaced. *Post-hoc* tests showed that this stronger increase in horizontal curvature when participants were not displaced was only apparent in the condition in which participants used a rod of 20 cm (*p* < 0.05). No other effects were significant.

Thus, the lengthening of the end-effector, and, for horizontal curvature, the displacement of the participant, affected the kinematics of the pointing movements, which is in agreement with previous results on tool use kinematics, for the effects of rod length, respectively (Dean and Brüwer, 1997; Baird et al., 2002). Note that the values we found for the end-point kinematics were in line with results reported in other studies on reaching without a tool (Gordon et al., 1994; Haggard and Richardson, 1996; Desmurget et al., 1997).

### Joint-angle trajectories

**Figure 3:**
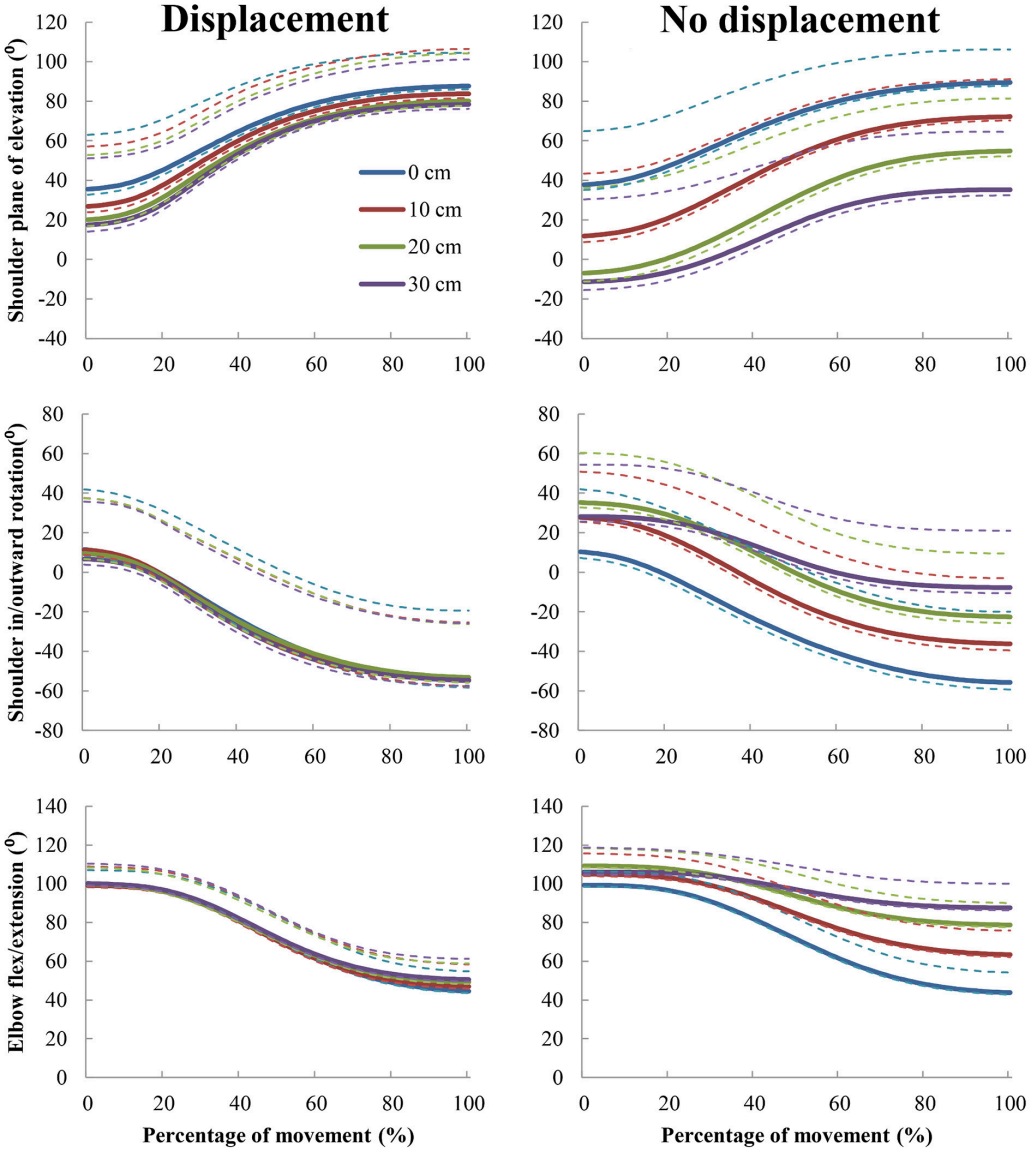
**Joint-angle trajectories of the three joint-angles with the largest contribution to the movement**. The dotted lines above the solid lines reflect the within-participants' standard deviation averaged across participants, the dotted lines below the solid lines reflect the standard error of the mean.

The trajectories of the three joint-angles shoulder plane of elevation, shoulder inward-outward rotation, and elbow flexion-extension (Figure 3) contributed most to the performed pointing movements (Supplementary Material), and are therefore discussed here (significant effects are presented in Table 3). Not surprisingly, all of these three angles changed during the unfolding of the movement, as demonstrated by large effects of movement instant. Over the course of the movement, the plane of elevation increased (i.e., the arm moved more forward), the shoulder angle turned outward, and the elbow angle extended. *Post-hoc* comparisons indicated that all instants differed significantly from each other for all selected angles (all *p*'s < 0.001).

**Table 3:** **Significant main and interaction effects for the selected joint-angles**.

**Dependent variable**	**Within-subject factor**		**Mean**	***SD***	***F***	***df***	***p***	**$\eta_{\text{G}}^{2}$**
**SHOULDER PLANE OF ELEVATION (**^0^**)**								
	Rod length	0 cm	67.08	29.37	68.97	1.18, 16.50	<0.001	0.18
		10 cm	53.57	34.22				
		20 cm	41.48	41.21				
		30 cm	34.18	42.12				
	Participant displacement	No	38.89	41.67	128.64	1, 14	<0.001	0.13
		Yes	59.26	33.35				
	Movement instant	1%	16.41	37.01	114.95	1.18, 16.57	<0.001	0.41
		25%	30.74	35.47				
		50%	55.97	31.71				
		75%	69.53	28.46				
		100%	72.73	28.08				
	Rod length × Participant displacement				63.20	1.76, 24.59	<0.001	0.08
**SHOULDER INWARD-OUTWARD ROTATION (**^0^**)**								
	Rod length	0 cm	−26.77	41.76	13.54	1.40, 19.59	0.001	0.05
		10 cm	−17.64	39.19				
		20 cm	−11.45	39.69				
		30 cm	−10.06	39.13				
	Participant displacement	No	−5.62	40.24	120.92	1, 14	<0.001	0.12
		Yes	−27.33	39.41				
	Movement instant	1%	17.58	28.64	99.25	1.18, 16.51	<0.001	0.39
		25%	3.24	31.84				
		50%	−22.67	34.88				
		75%	−38.08	34.42				
		100%	−42.46	35.06				
	Rod length × Participant displacement				45.01	1.89, 26.43	<0.001	0.06
**ELBOW FLEXION-EXTENSION (**^0^**)**								
	Rod length	0 cm	71.93	24.11	34.87	1.79, 25.08	<0.001	0.21
		10 cm	78.41	22.97				
		20 cm	83.70	22.04				
		30 cm	85.67	22.01				
	Participant displacement	No	86.79	21.44	522.36	1, 14	<0.001	0.30
		Yes	73.07	23.22				
	Movement instant	1%	102.25	10.03	1088.65	2.065, 28.91	<0.001	0.75
		25%	97.67	11.03				
		50%	78.86	15.44				
		75%	62.77	18.00				
		100%	58.10	19.09				
	Rod length × Participant displacement				67.61	3, 42	<0.001	0.16
	Rod length × Movement instant				73.21	3.38, 47.24	<0.001	0.08
	Participant displacement × Movement instant				126.26	1.47, 20.59	<0.001	0.08
	Rod length × Participant displacement × Movement instant				69.50	2.38, 33.67	<0.001	0.05

Furthermore, all three selected angles showed significant effects for rod length, in which the plane of elevation came closer to the frontal plane, the shoulder angle turned more inward, and the elbow angle was more flexed when participants used a longer rod. *Post-hoc* analysis showed that the plane of elevation angle differed significantly across the different rod lengths (all *p*'s < 0.001). Furthermore, the inward-outward rotation angle of the shoulder differed only between index finger condition and all rod conditions (all *p*'s < 0.01). With the exception of the 20 and 30 cm rod, the flexion-extension angle of the elbow differed across all rod lengths (all *p*'s < 0.01). However, these effects of changing angles with longer rod lengths generally stem from the conditions in which the participant was not displaced, as indicated by the significant interactions between rod length and participant placement for all selected joint-angles. In these effects, the abovementioned changes in used joint-angles were apparent when the rod length increased and the participant was not displaced, whereas when the participant was displaced, practically the same joint-angle trajectories were used across all rod lengths (Figure 3). *Post-hoc* comparisons supported these effects, with the abovementioned directions, across all rod lengths for all angles (all *p*'s < 0.001). This was with the exception of movements with the index finger, which was in essence the same condition between displacement and no displacement conditions. This pattern in the data is also supported by the effect of participant placement, in which similar directions of effects on the used joint-angles, as found in the effects on rod length, were found when participants were not displaced.

Finally, there were some small interaction effects regarding the elbow angle. First, an interaction between rod length and movement instant showed that the elbow angle extended less across the movement when the rod length increased. Indeed, *post-hoc* tests revealed that the elbow angle only differed significantly across rod lengths from the third instant till the end of the movement, with the exception of the difference in elbow angle between the 10 and 20 cm, 10 and 30 cm, and 20 and 30 cm rods, which was significant from the fourth instant till the end of the movement (all significant *p*'s < 0.001). This reduced extension was also apparent across the movement when the participant was not displaced, although it was a small effect. All *post-hoc* comparisons between the displacement and no displacement condition were significant at all instants (all *p*'s < 0.001). The three-way interaction between rod length, participant placement, and movement instant showed that the above-mentioned interactions were predominantly present when the participant was not displaced (Figure 3). *Post-hoc* comparisons supported this conclusion, showing that the elbow angle did not differ significantly across rod lengths for different instances when the participant was displaced, whereas the elbow angle differed across rods over the movement from the second instant till the end of the movement. This was with exception of the differences in elbow angle between the 10 and 20 cm, and 10 and 30 cm rods, which were significant from the third instant till the end of the movement when participants were not displaced, and the difference in elbow angle between the 20 and 30 cm rods, which was significant from the fourth instant till the end of the movement when participants were not displaced (all significant *p*'s < 0.001). No other effects were significant for these three selected angles.

Summarizing the effects of joint-angle trajectories, it can be stated that movements of the selected joint-angles were remarkably similar across rod lengths when participants were displaced with respect to the target in accordance with the length of the rod. Importantly, participants used different joint-angle trajectories when they were not displaced. These findings supported our hypothesis.

### Joint-angle coordination patterns

The question remained how joint-angle coordination patterns were organized when participants used these different solutions in joint-angle trajectories across different rod lengths. This was examined by analyzing the UCM variables NGEV_Log_, GEV_Log_, and the ratio between these variables, V_RatioLog_.

**Figure 4:**
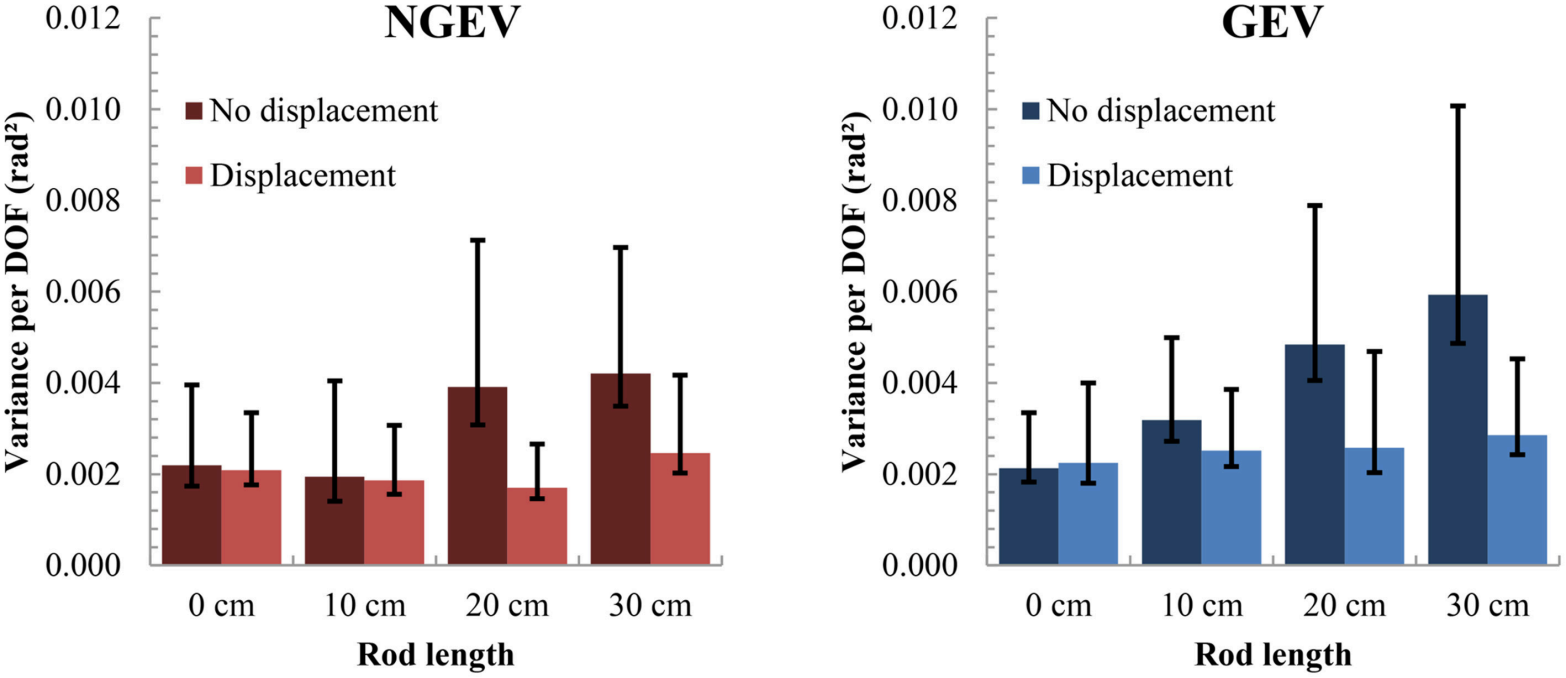
**Distributions of GEV and NGEV, across different rod lengths for both displacement and no-displacement conditions**. Upper error bars reflect the standard deviation across participants; lower error bars reflect the standard error of the mean. Note that the statistical analysis was performed on the log transformed GEV (GEV_Log_) and NGEV (NGEV_Log_).

Results showed that as longer rods were used, values of NGEV_Log_ became larger as well [mean [SD]: 0 cm: −6.43 [0.83], 10 cm: −6.57 [0.81], 20 cm: −6.18 [0.78], 30 cm: −6.01 [0.87]; *F*_(3, 42)_ = 5.19, *p* < 0.005, $\eta_{\text{G}}^{2}$ = 0.07]. Furthermore, NGEV_Log_ increased more when the rod length was longer in conditions in which the participant was not displaced [*F*_(3, 42)_ = 3.73, *p* < 0.05, $\eta_{\text{G}}^{2}$ = 0.05; Figure 4]. Bonferroni-corrected paired *t*-tests between the two displacement conditions for each rod length separately revealed that only for pointing movements with the rod of 20 cm values of NGEV_Log_ were increased significantly in when participants were not displaced (−5.82 [0.77]) as compared with the condition in which participants were displaced (−6.54 [0.64], *t*_14_ = 3.04, *p* < 0.01). Although, *post-hoc* tests revealed only significant differences between values of NGEV_Log_ between displacement conditions for the 20 cm rod, Figure 4, in line with the above-mentioned interaction, shows a trend for larger differences in values of NGEV_Log_ between displacements conditions for longer rods. The lack of a significant *post-hoc* test for the longest rod might be because inter-participant variability was higher in those conditions in which the longest rod was used, as indicated by large standard errors of the mean (Figure 4). Note that participant placement had no significant effect on NGEV_Log._

Also GEV_Log_ increased as the rod length increased [mean [SD]: 0 cm: −6.28 [0.54], 10 cm: −6.01 [0.56], 20 cm: −5.85 [0.71], 30 cm: −5.73 [0.81]; *F*_(1.86, 26.06)_ = 7.00, *p* < 0.005, $\eta_{\text{G}}^{2}$ = 0.10]. Furthermore, GEV_Log_ was higher in conditions in which the participant was not displaced in comparison with conditions in which the participant was displaced [not displaced: −5.78 [0.74]; displaced: −6.15 [0.58]; *F*_(1, 14)_ = 10.34, *p* < 0.01, $\eta_{\text{G}}^{2}$ = 0.08]. Moreover, GEV_Log_ increased more when the rod length increased in conditions in which the participant was not displaced [*F*_(3, 42)_ = 3.13, *p* < 0.05, $\eta_{\text{G}}^{2}$ = 0.05; Figure 4], especially for the longer rods. Bonferroni-corrected paired *t*-tests between the two displacement conditions for each rod length separately showed that values of GEV_Log_ were higher for movements with the 20 cm rod in the situation, in which participants were not displaced (−5.42 [0.63]) as compared with the situation, in which participants were displaced (−6.18 [0.64], *t*_14_ = 3.94, *p* < 0.001). As with the *post-hoc* tests on displacement conditions with respect to NGEV_Log_, there is a trend visible (Figure 4), with higher values of GEV_Log_ in conditions with longer rods when participants were not displaced, as compared with the conditions in which participants were displaced. Again, the large variability between participants in conditions with longer rods, and thus large standard errors of the means, might be the reason for the lack of significant *post-hoc* tests.

No effects on the UCM variable V_RatioLog_ were found. This indicates that GEV_Log_ and NGEV_Log_ increased more or less equally across the different conditions, causing only little, non-significant, changes in the value of V_RatioLog_. Importantly, for all conditions, V_RatioLog_ was larger than zero (mean [SD]: 0.33 [0.70]), demonstrating that in all conditions GEV_Log_ was larger than NGEV_Log_ (see Figure 4), suggesting stable performance of the task at hand^1^.

From these results, it can be concluded that both NGEV_Log_ and GEV_Log_ increased when the rod length increased, especially in the conditions in which the participants were not displaced. Since this increase in both NGEV_Log_ and GEV_Log_ was similar, the values of V_RatioLog_ stayed more or less the same across conditions. Participants proved to be able to maintain the flexible coordination in joint-angle trajectories, that is, a higher value of GEV relative to NGEV, to stabilize the performance variable, even when the use of tools induced the use of different joint-angle coordination patterns in the movement.

## Discussion

The current study focused on how body and tool function as one system. Therefore, point-to-point movements with and without a rod attached to the index finger, with and without participants' displacement, were examined. The length of the rod was systematically varied and participants were either displaced from the target with a distance equal to the length of the rod, or the distance to the target was kept the same across different rod lengths. The results generally confirmed our hypotheses; the end-effector kinematics were affected by rod length; for longer rods, the tip movements had a longer deceleration time, a higher peak velocity, more curvature in the horizontal plane, and were lifted higher. In addition, the end-effector movement was more curved in the horizontal plane when participants were not displaced. When participants were not displaced, joint-angle trajectories were affected such that, with longer rods, the shoulder plane of elevation came closer to the frontal plane, the shoulder angle turned more inward, and the elbow angle was more flexed. Interestingly, both the variability in the joints affecting end-effector position and the variability not affecting end-effector position increased with rod length, while this increase was larger for one of the longest rods when participants were not displaced. The ratio between these measurements of variability did not differ among conditions, suggesting a similar degree of stabilization independent of rod length and participant placement. These findings showed that the specifics of the tool (i.e., its length) and the task (i.e., how much room there is for the additional length) determined the specifics of the kinematics of the movement, and the joint-angle trajectories, with which the rod was moved. Importantly, the stabilization of the rod tip was not affected by these differences, suggesting that body and tool functioned as one system in all situations.

The stabilization of the end-effector position by the body-plus-tool system implies that kinematic characteristics of the end-effector movement can vary, as long as it is ensured that the tip movement is stabilized by covariation in joint-angles. In other words, the stabilization of the end-effector movement allows for different ways in which the actions of the body-plus-tool system are performed. This means that kinematic characteristics of the trajectory can be affected by mechanical properties of the rod, such as its moment of inertia. Moreover, depending on the situation and the task (e.g., participant placement, target location, and length of the rod), joint-angles may be adapted to produce the movement. Importantly, both effects were found in the current study. We found that the end-effector movement had a higher peak velocity, a longer deceleration time, and was more curved when the end-effector increased. Moreover, not displacing participants resulted in more horizontal curvature of the tip movement. The joint-angle trajectories were adjusted depending on whether the participant was displaced or not. Importantly, in all conditions, these movements were stabilized to a similar extent by the body-plus-tool system, since the values of V_RatioLog_ remained indifferent across these conditions.

The present findings for differences in kinematic characteristics of pointing movements, when end-effector length is increased, are in good agreement with earlier work on tool use kinematics (Dean and Brüwer, 1997; Baird et al., 2002). However, whereas Dean and Brüwer (1997) found smaller movement times with longer probe lengths, we found, in accordance with Baird et al. (2002), longer movement times with longer rod lengths. This contradiction can be explained by the fact that Dean and Brüwer (1997) studied 2-D pointing movements with a manipulandum, mostly based on rotations. With longer probes, small rotations of the hand can result in large movements at the tip of the probe, resulting in shorter movement times. The movements of the current study did not contain this kind of rotatory movements, which could explain why we, in agreement with Baird et al. (2002), found increasing movement times as rod length increased. We extended these findings in that we found that the increase in movement times with longer rods stemmed from increase of deceleration time. The longer deceleration times are generally considered to follow from feedback processes (Elliott et al., 2001, 2010). Moreover, we showed that movements with longer rods had higher curvature in the horizontal plane; so despite the fact that, in the process of learning to use a tool, the end-effector trajectory becomes straighter (Heuer and Sülzenbrück, 2009), the length of the tool does influence curvature. Finally, we showed that the tip of longer rods was lifted higher than that of shorter rods. This might be due to the simple fact that for long rods a small upward movement of the wrist can result in a large upward movement in the tip. In addition, the effects as found in this study could also result from the additional weight included by the lengthening of the rods. The fact that after exercise, the positional matching of joint-angles with a reference arm is perturbed (Walsh et al., 2004, 2006), suggests that the perceived effort, which increases after exercise, influences the way in which joint-angles are perceived. This means that changing the length of the rod, which includes changes in mass and changes in moment of inertia, could have altered the perceived effort during the pointing movements, thereby contributing to the effects as reported in this study.

Our finding that there was very little difference in both joint-angle trajectories and joint-angle coordination patterns across the different end-effector lengths when participants were displaced from the target, as the end-effector length increased, is in agreement with findings of Van der Steen and Bongers (2011). This reflects the fact that, as expected, participants used similar joint-angle trajectories and joint-angle coordination patterns, when this would result in adequate task performance across the different rods. Additionally, participants used different joint-angle trajectories across different end-effector lengths to perform the pointing movements when they were not displaced, in accordance with previous findings on joint-angle trajectories in tool use (Cruse et al., 1990; Dean and Brüwer, 1997). This is not surprising; an increasing end-effector length that is not counteracted by a displacement challenged the participants to find other solutions for the task at hand, at least in terms of joint-angle trajectories. Interestingly, participants not only changed their joint-angle trajectories but also the joint-angle coordination patterns underlying the stabilization of the end-effector movement. That is, GEV and NGEV both increased with rod length. Importantly, independent of these changes in joint-angle variability patterns, the stabilization of the end-effector was similar across conditions.

We found that participants increased variability in joint-angles as rod length increased. This is in accordance with changes in variability of joint-angles during stone flaking, which increased as the size of the flake increased (Rein et al., 2013). Although, the increase in length of the body-plus-tool system with the flakes is, of course, much smaller than with our manipulation of rod length, in both studies the size of the tool was varied. The use of larger tools alters the moments of inertia of the end-effector of the body-plus-tool system and requires the use of more extreme joint-angles, which may induce additional noise to the system. This additional noise could be the cause of the increase in NGEV (cf. Greve et al., 2015) when tool size increases, because extra noise in the neuromotor system affects the stability at the tip of the tool. Moreover, an equivalent increase in GEV with increasing tool lengths, as found in the current study, preserves stable behavior during the tooling movements. Thus, both the increase in NGEV due to additional noise and the necessary increase in GEV to preserve stable movement contributed to the increase in joint-angle variability when using larger tools. Employing the strategy to increase joint-angle covariation to counteract errors induced at the tip of the end-effector ensures the preservation of movement accuracy despite changes in characteristics of the body-plus-tool system. This strategy, that is, preserving values of GEV higher than NGEV, have been shown before in studies on joint-angle coordination during pointing tasks (Scholz et al., 2000; Domkin et al., 2002, 2005; Tseng et al., 2002, 2003; Van der Steen and Bongers, 2011; Kim et al., 2012; Krüger et al., 2012; Verrel et al., 2012; Rein et al., 2013; Greve et al., 2015) and support the findings of the current study.

The conclusion that during tool use the body and tool are united in a body-plus-tool system can be linked to previous studies on tool use. Specifically, several studies suggested that during the use of a tool, the control of the end-effector movement shifts from the hand to the tool (Bongers et al., 2003, 2004; Arbib et al., 2009; Jacobs et al., 2009; Van Elk et al., 2011). For instance, during the movements with a sliding lever that had a complex mapping between the hand and the tip of the lever, the trajectory of the lever's tip was straighter than the path of the hand, which suggested that the lever's tip was the newly controlled end-effector (Heuer and Sülzenbrück, 2009). In addition, when participants had to imitate different grasping movements, as represented in a picture showing the required action with either hand or tool, reaction times of hand and tool were not affected by the effector type shown in the picture (Van Elk et al., 2011). In other words, responding to a tool picture took as long as responding to a non-tool picture, both for grasping movements with the hand or the tool. This suggested that imitation of hand or tool actions are effector-independent, indicating that hand or tool actions are organized in a similar way. In total, the above-mentioned studies suggest, in accordance with the findings of the current paper, that the tool is incorporated in the body-plus-tool system during tool use.

Furthermore, studies on a neural level support the idea of a body-plus-tool system that stabilizes the end-effector kinematics during tool use. For example, similar cortical motor neurons in monkeys became active both when grasping with the hand and grasping with pliers, as if the pliers had become the fingers of the hand (Umiltà et al., 2008). In addition, grip planning in hand and tool conditions after training was associated with similar increases in activity within the same regions of the anterior intraparietal and caudal ventral premotor cortices (Jacobs et al., 2010). Moreover, reactions in the left lateral occipitotemporal cortex closely overlapped both tool and hand actions (Bracci et al., 2012). All of these studies suggest the employment of the plasticity of the central nervous system to switch from a body-without-tool system toward a body-plus-tool system in order to achieve the task at hand. In line with this reasoning, kinematics of free-hand movements and perception of length of the arm are altered directly after the use of tools (Cardinali et al., 2009), as if the switch from body-plus-tool system to body-without-tool system takes some time.

In conclusion, the current study has shown that the stabilization of the end-effector movements is coordinated in a similar way across actions with tools of different lengths and the index finger, despite the use of different kinematics, joint-angle trajectories, and coordination patterns. This suggests that during tool use, the body and tool become one system to stabilize the end-effector movement at hand.

## Author contributions

RB, TV designed the study, TV conducted the experiment and performed the analysis. TV, RB wrote the programs to run the data analysis. TV, RB, and LM interpreted the results, wrote the manuscript and contributed to the writing at all stages. All authors confirmed on the final version of the paper.

### Conflict of interest statement

The authors declare that the research was conducted in the absence of any commercial or financial relationships that could be construed as a potential conflict of interest.

## Footnotes

^1^We examined whether the effects of the UCM measures stemmed from multi-joint covariation and not from individual joint-angle variability (Yen and Chang, 2010; Verrel, 2011). Therefore, we repeated the UCM analyses as before but now with a covariance matrix in which all covariation among joint-angles was removed by setting the off-diagonal terms of the original covariance matrix to zero. The results demonstrated that the UCM effects as reported originate from multi-joint covariation.

## References

[B1] Arbib M. A. Bonaiuto J. B. Jacobs S. Frey S. H. (2009). Tool use and the distalization of the end-effector. Psychol. Res. 73, 441–462. 10.1007/s00426-009-0242-2 19347356 PMC2734956

[B2] Baird K. M. Hoffmann E. R. Drury C. G. (2002). The effects of probe length on Fitts' law. Appl. Ergon. 33, 9–14. 10.1016/S0003-6870(01)00049-7 11827141

[B3] Bakeman R. (2005). Recommended effect size statistics for repeated measures designs. Behav. Res. Methods 37, 379–384. 10.3758/BF03192707 16405133

[B4] Bongers R. M. Michaels C. F. Smitsman A. W. (2004). Variations of tool and task characteristics reveal that tool-use postures are anticipated. J. Mot. Behav. 36, 305–315. 10.3200/JMBR.36.3.305-315 15262626

[B5] Bongers R. M. Smitsman A. W. Michaels C. F. (2003). Geometrics and dynamics of a rod determine how it is used for reaching. J. Mot. Behav. 35, 4–22. 10.1080/00222890309602117 12724095

[B6] Bracci S. Cavina-Pratesi C. Ietswaart M. Caramazza A. Peelen M. V. (2012). Closely overlapping responses to tools and hands in left lateral occipitotemporal cortex. J. Neurophysiol. 107, 1443–1456. 10.1152/jn.00619.2011 22131379

[B7] Cardinali L. Frassinetti F. Brozzoli C. Urquizar C. Roy A. C. Farnè A. (2009). Tool-use induces morphological updating of the body schema. Curr. Biol. 19, R478–R479. 10.1016/j.cub.2009.05.009 19549491

[B8] Cohen J. (1988). Statistical Power Analysis for the Behavioral Sciences, 2nd Edn. Hillsdale, NJ: Erlbaum.

[B9] Cruse H. Wischmeyer E. Brüwer M. Brockfeld P. Dress A. (1990). On the cost functions for the control of the human arm movement. Biol. Cybern. 62, 519–528. 10.1007/BF00205114 2357475

[B10] Dean J. Brüwer M. (1997). Control of human arm movements in two dimensions: influence of pointer length on obstacle avoidance. J. Mot. Behav. 29, 47–63. 10.1080/00222899709603469 20037009

[B11] Desmurget M. Jordan M. Prablanc C. Jeannerod M. (1997). Constrained and unconstrained movements involve different control strategies. J. Neurophysiol. 77, 1644–1650. 9084629 10.1152/jn.1997.77.3.1644

[B12] Domkin D. Laczko J. Djupsjöbacka M. Jaric S. Latash M. L. (2005). Joint angle variability in 3D bimanual pointing: uncontrolled manifold analysis. Exp. Brain Res. 163, 44–57. 10.1007/s00221-004-2137-1 15668794

[B13] Domkin D. Laczko J. Jaric S. Johansson H. Latash M. L. (2002). Structure of joint variability in bimanual pointing tasks. Exp. Brain Res. 143, 11–23. 10.1007/s00221-001-0944-1 11907686

[B14] Elliott D. Hansen S. Grierson L. E. M. Lyons J. Bennett S. J. Hayes S. J. (2010). Goal-directed aiming: two components but multiple processes. Psychol. Bull. 136, 1023–1044. 10.1037/a0020958 20822209

[B15] Elliott D. Helsen W. F. Chua R. (2001). A century later: Woodworth's (1899) two-component model of goal-directed aiming. Psychol. Bull. 127, 342–357. 10.1037/0033-2909.127.3.342 11393300

[B16] Gibson J. J. (2015). The Ecological Approach to Visual Perception. New York, NY: Psychology Press.

[B17] Gordon J. Ghilardi M. F. Cooper S. E. Ghez C. (1994). Accuracy of planar reaching movements. II. Systematic extent errors resulting from inertial anisotropy. Exp. Brain Res. 99, 112–130. 10.1007/BF00241416 7925785

[B18] Greve C. Hortobàgyi T. Bongers R. M. (2015). Physical demand but not dexterity is associated with motor flexibility during rapid reaching in healthy young adults. PLoS ONE 10:e127017. 10.1371/journal.pone.0127017 25970465 PMC4430491

[B19] Haggard P. Richardson J. (1996). Spatial patterns in the control of human arm movement. J. Exp. Psychol. 22, 42–62. 10.1037/0096-1523.22.1.42 8742251

[B20] Heuer H. Sülzenbrück S. (2009). Trajectories in operating a handheld tool. J. Exp. Psychol. 35, 375–389. 10.1037/0096-1523.35.2.375 19331495

[B21] Jacobs S. Bussel B. Combeaud M. Roby-Brami A. (2009). The use of a tool requires its incorporation into the movement: evidence from stick-pointing in apraxia. Cortex 45, 444–455. 10.1016/j.cortex.2007.12.009 19231475

[B22] Jacobs S. Danielmeier C. Frey S. H. (2010). Human anterior intraparietal and ventral premotor cortices support representations of grasping with the hand or a novel tool. J. Cogn. Neurosci. 22, 2594–2608. 10.1162/jocn.2009.21372 19925200

[B23] Kim M. J. Karol S. Park J. Auyang A. Kim Y. H. Kim S.. (2012). Inter-joint synergies increase with motor task uncertainty in a whole-body pointing task. Neurosci. Lett. 512, 114–117. 10.1016/j.neulet.2012.01.072 22343023

[B24] Krüger M. Borbély B. Eggert T. Straube A. (2012). Synergistic control of joint angle variability: influence of target shape. Hum. Mov. Sci. 31, 1071–1089. 10.1016/j.humov.2011.12.002 22244105

[B25] Latash M. L. Scholz J. P. Schöner G. (2007). Toward a new theory of motor synergies. Motor Control 11, 276–308. 17715460 10.1123/mcj.11.3.276

[B26] Olejnik S. Algina J. (2003). Generalized eta and omega squared statistics: measures of effect size for some common research designs. Psychol. Methods 8, 434–447. 10.1037/1082-989X.8.4.434 14664681

[B27] Rein R. Bril B. Nonaka T. (2013). Coordination strategies used in stone knapping. Am. J. Phys. Anthropol. 150, 539–550. 10.1002/ajpa.22224 23359287

[B28] Scholz J. P. Schöner G. (1999). The uncontrolled manifold concept: identifying control variables for a functional task. Exp. Brain Res. 126, 289–306. 10.1007/s002210050738 10382616

[B29] Scholz J. P. Schöner G. Latash M. L. (2000). Identifying the control structure of multijoint coordination during pistol shooting. Exp. Brain Res. 135, 382–404. 10.1007/s002210000540 11146817

[B30] Schöner G. (1995). Recent developments and problems in human movement science and their conceptual implications. Ecol. Psychol. 12, 291–314. 10.1207/s15326969eco0704_5

[B31] Tseng Y.-W. Scholz J. P. Schöner G. (2002). Goal-equivalent joint coordination in pointing: affect of vision and arm dominance. Motor Control 6, 183–207. 12122226 10.1123/mcj.6.2.183

[B32] Tseng Y.-W. Scholz J. P. Schöner G. Hotchkiss L. (2003). Effect of accuracy constraint on joint coordination during pointing movements. Exp. Brain Res. 149, 276–288. 10.1007/s00221-002-1357-5 12632230

[B33] Umiltà M. A. Escola L. Intskirveli I. Grammont F. Rochat M. Caruana F.. (2008). When pliers become fingers in the monkey motor system. Proc. Natl. Acad. Sci. U.S.A. 105, 2209–2213. 10.1073/pnas.0705985105 18238904 PMC2538900

[B34] Van Andel C. J. Wolterbeek N. Doorenbosch C. A. M. Veeger D. H. E. J. Harlaar J. (2008). Complete 3D kinematics of upper extremity functional tasks. Gait Posture 27, 120–127. 10.1016/j.gaitpost.2007.03.002 17459709

[B35] Van der Steen M. C. Bongers R. M. (2011). Joint angle variability and co-variation in a reaching with a rod task. Exp. Brain Res. 208, 411–422. 10.1007/s00221-010-2493-y 21127846 PMC3018264

[B36] Van Elk M. Van Schie H. T. Bekkering H. (2011). Imitation of hand and tool actions is effector-independent. Exp. Brain Res. 214, 539–547. 10.1007/s00221-011-2852-3 21904930 PMC3183242

[B37] Verrel J. (2010). Distributional properties and variance-stabilizing transformations for measures of uncontrolled manifold effects. J. Neurosci. Methods 191, 166–170. 10.1016/j.jneumeth.2010.06.016 20599556

[B38] Verrel J. (2011). A formal and data-based comparison of measures of motor-equivalent covariation. J. Neurosci. Methods 200, 199–206. 10.1016/j.jneumeth.2011.04.006 21513738

[B39] Verrel J. Lövdén M. Lindenberger U. (2012). Normal aging reduces motor synergies in manual pointing. Neurobiol. Aging 33, 200.e1–200.e10. 10.1016/j.neurobiolaging.2010.07.006 20724037

[B40] Walsh L. D. Allen T. J. Gandevia S. C. Proske U. (2006). Effect of eccentric exercise on position sense at the human forearm in different postures. J. Appl. Physiol. 100, 1109–1116. 10.1152/japplphysiol.01303.2005 16373445

[B41] Walsh L. D. Hesse C. W. Morgan D. L. Proske U. (2004). Human forearm position sense after fatigue of elbow flexor muscles. J. Physiol. 558, 705–715. 10.1113/jphysiol.2004.062703 15181165 PMC1664958

[B42] Wu G. Van der Helm F. C. T. Veeger H. E. J. D. Makhsous M. Van Roy P. Anglin C.. (2005). ISB recommendation on definitions of joint coordinate systems of various joints for the reporting of human joint motion–Part II: shoulder, elbow, wrist and hand. J. Biomech. 38, 981–992. 10.1016/j.jbiomech.2004.05.042 15844264

[B43] Yen J. T. Chang Y.-H. (2010). Rate-dependent control strategies stabilize limb forces during human locomotion. J. R. Soc. Interface 7, 801–810. 10.1098/rsif.2009.0296 19828502 PMC2874235

