# Supplementary material for: Joint-Angle Coordination Patterns Ensure Stabilization of a Body-Plus-Tool System in Point-to-Point Movements with a Rod
Source: Front Psychol. 2016 Jun 3;7:826. doi: 10.3389/fpsyg.2016.00826 (PMC4891357; doi:10.3389/fpsyg.2016.00826)
Supplement: Supplementary file 1 [file Table1.pdf]

Supplementary Material: Supplementary Table regarding all effects of analyzed joint-angles

Joint-angle coordination patterns ensure stabilization of a body-plus-tool system in point-to-point movements with a rod

Tim A. Valk[1*], Leonora J. Mouton[1], Raoul M. Bongers[1]

[1]University of Groningen, University Medical Center Groningen, Center for Human Movement Sciences, Groningen, the Netherlands

*** Correspondence:**
Tim A. Valk
tim_valk@hotmail.com

Table S1. Full table of all significant main and interaction effects of the analyzed joint-angles.

Dependent variable	Within-subject factor		Mean	SD	*F*	*df*	*p*	η^2_G
Shoulder elevation (^{o})								
	End-effector length x Movement instant				28.76	1.62;22.70	<0.001	0.04
	Participant displacement x Movement instant				34.90	1.11;15;52	<0.001	0.05
	End-effector length x Participant displacement x Movement instant				20.70	1.81;25.40	<0.001	0.03
Shoulder plane of elevation (^{0})								
	End-effector length	0 cm	67.08	29.37	68.97	1.18;16.50	<0.001	0.18
		10 cm	53.57	34.22				
		20 cm	41.48	41.21				
		30 cm	34.18	42.12				
	Participant displacement	No	38.89	41.67	128.64	1;14	<0.001	0.13
		Yes	59.26	33.35				
	Movement instant	1%	16.41	37.01	114.95	1.18;16.57	<0.001	0.41
		25%	30.74	35.47				
		50%	55.97	31.71				
		75%	69.53	28.46				
		100%	72.73	28.08				
	End-effector length x Participant displacement				63.20	1.76;24.59	<0.001	0.08
Shoulder inward-outward rotation (^{0})								
	End-effector length	0 cm	-26.77	41.76	13.54	1.40;19.59	0.001	0.05
		10 cm	-17.64	39.19				
		20 cm	-11.45	39.69				
		30 cm	-10.06	39.13				
	Participant displacement	No	-5.62	40.24	120.92	1;14	<0.001	0.12
		Yes	-27.33	39.41				
	Movement instant	1%	17.58	28.64	99.25	1.18;16.51	<0.001	0.39
		25%	3.24	31.84				
		50%	-22.67	34.88				
		75%	-38.08	34.42				
		100%	-42.46	35.06				
	End-effector length x Participant displacement				45.01	1.89;26.43	<0.001	0.06
Elbow flexion-extension (^{0})								
	End-effector length	0 cm	71.93	24.11	34.87	1.79;25.08	<0.001	0.21
		10 cm	78.41	22.97				
		20 cm	83.70	22.04				
		30 cm	85.67	22.01				
	Participant displacement	No	86.79	21.44	522.36	1;14	<0.001	0.30
		Yes	73.07	23.22				
	Movement instant	1%	102.25	10.03	1088.65	2.065;28.91	<0.001	0.75
		25%	97.67	11.03				
		50%	78.86	15.44				
		75%	62.77	18.00				
		100%	58.10	19.09				
	End-effector length x Participant displacement				67.61	3;42	<0.001	0.16
	End-effector length x Movement instant				73.21	3.38;47.24	<0.001	0.08
	Participant displacement x Movement instant				126.26	1.47;20.59	<0.001	0.08
	End-effector length x Participant displacement x Movement instant				69.50	2.38;33.67	<0.001	0.05

Table S1 resumed. Full table of all significant main and interaction effects of the analyzed joint-angles.

Dependent variable	Within-subject factor		Mean	SD	*F*	*df*	*p*	η^2_G
Forearm pronation-supination (°)								
	End-effector length	0 cm	140.70	10.58	13.07	1.77;24;75	<0.001	0.11
		10 cm	131.48	16.79				
		20 cm	127.23	20.16				
		30 cm	125.37	22.28				
	Participant displacement	No	128.89	20.08	11.07	1;14	0.005	0.02
		Yes	133.50	170.41				
	Movement instant	1%	129.66	19.67	64.19	1.58;22.08	<0.001	0.06
		25%	125.87	19.06				
		50%	128.69	18.06				
		75%	134.21	17.58				
		100%	137.54	18.10				
	End-effector length x Participant displacement				4.61	1.73;24.22	<0.05	0.02
Wrist flexion-extension (°)								
	End-effector length	0 cm	6.86	10.34	16.71	1.78;24.92	<0.001	0.24
		10 cm	1.82	11.16				
		20 cm	-5.10	14.78				
		30 cm	-9.08	17.17				
	Participant displacement	No	-6.79	16.89	37.77	1;14	<0.001	0.20
		Yes	4.03	10.14				
	Movement instant	1%	-6.03	18.69	18.74	1.05;14.76	0.001	0.08
		25%	-4.07	17.00				
		50%	-0.09	13.19				
		75%	1.21	11.52				
		100%	2.10	11.34				
	End-effector length x Participant displacement				17.32	2.13;39.78	<0.001	0.12
	Participant displacement x Movement instant				29.73	1.08;15.12	<0.001	0.03
Wrist abduction-adduction (°)								
	End-effector length	0 cm	-16.68	7.20	16.00	3;42	<0.001	0.08
		10 cm	-20.91	7.93				
		20 cm	-21.77	7.50				
		30 cm	-22.40	8.63				
Finger abduction-adduction (°)								
	End-effector length	0 cm	21.30	7.98	7.33	1.57;22.00	0.006	0.04
		10 cm	17.85	7.90				
		20 cm	17.72	7.11				
		30 cm	20.33	6.91				
