# Supplementary material for: Joint-Angle Coordination Patterns Ensure Stabilization of a Body-Plus-Tool System in Point-to-Point Movements with a Rod
Source: Front Psychol. 2016 Jun 3;7:826. doi: 10.3389/fpsyg.2016.00826 (PMC4891357; doi:10.3389/fpsyg.2016.00826)
Supplement: Supplementary file 2 [file Image1.PDF]

Supplementary Material: Supplementary Figure of the trajectories regarding all analyzed joint-angles

Joint-angle coordination patterns ensure stabilization of a body-plus-tool system in point-to-point movements with a rod

Tim A. Valk[1*], Leonora J. Mouton[1], Raoul M. Bongers[1]

[1]University of Groningen, University Medical Center Groningen, Center for Human Movement Sciences, Groningen, the Netherlands

*** Correspondence:**
Tim A. Valk
tim_valk@hotmail.com

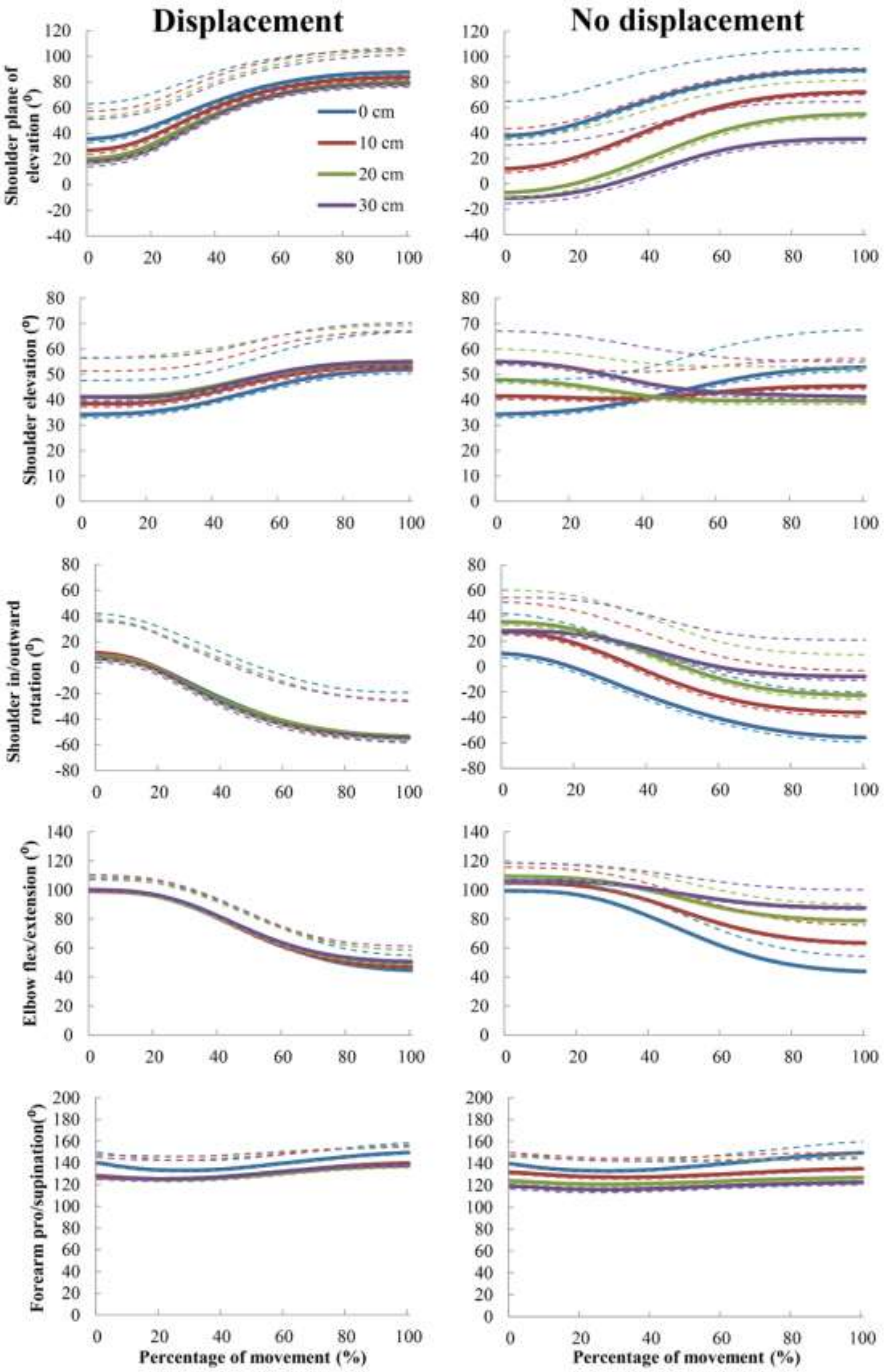

Figure S1. *Joint-angle trajectories of all joint-angles included in the study.* The dotted lines above the solid lines reflect the within-participants' standard deviation averaged across participants, the dotted lines below the solid lines reflect the standard error of the mean.

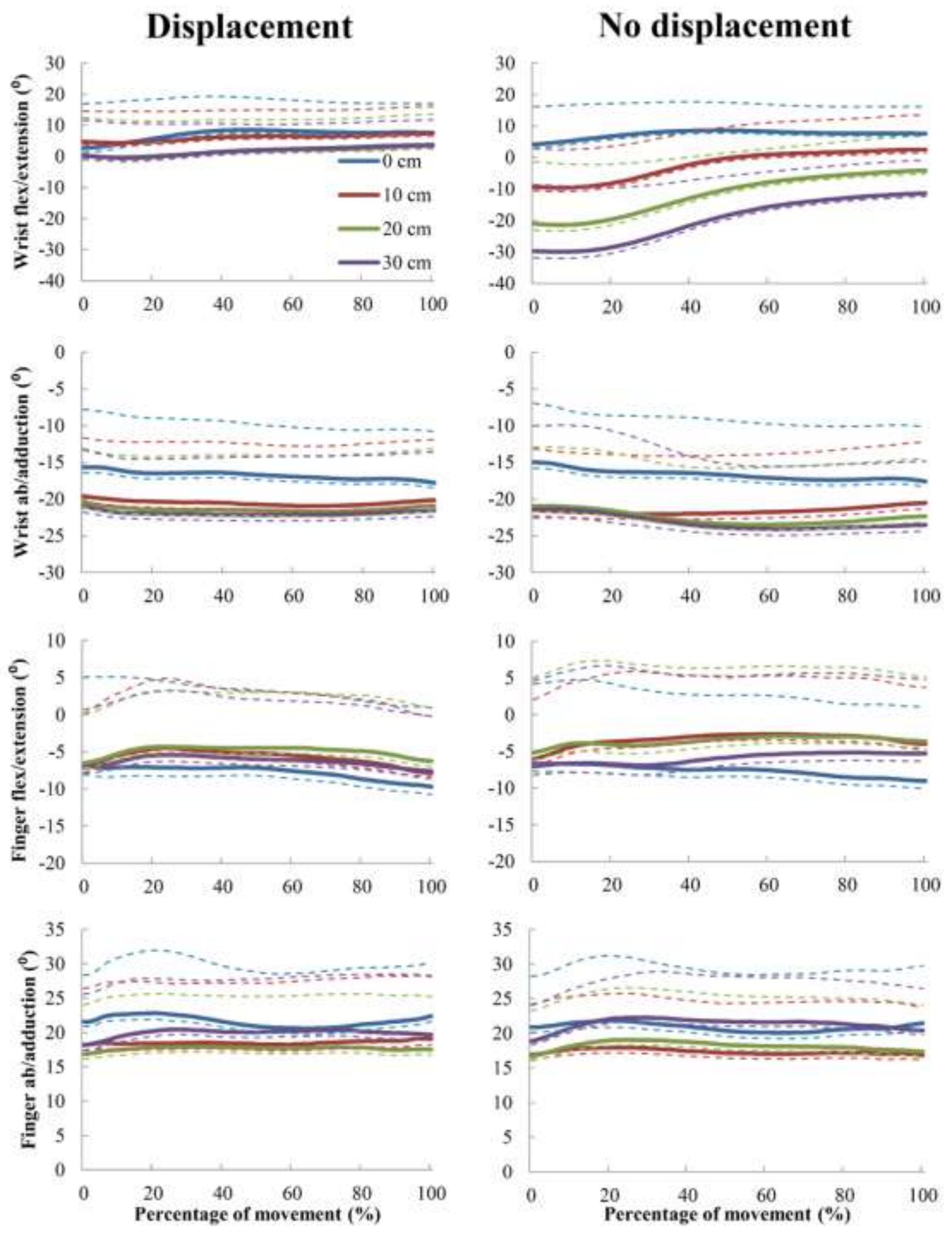

Figure S1 resumed. *Joint-angle trajectories of all joint-angles included in the study.* The dotted lines above the solid lines reflect the within-participants' standard deviation averaged across participants, the dotted lines below the solid lines reflect the standard error of the mean.
